# Evolutionary Origin of Distinct NREM and REM Sleep

**DOI:** 10.3389/fpsyg.2020.567618

**Published:** 2020-12-14

**Authors:** Risa Yamazaki, Hirofumi Toda, Paul-Antoine Libourel, Yu Hayashi, Kaspar E. Vogt, Takeshi Sakurai

**Affiliations:** ^1^CNRS UMR 5292, INSERM U1028, Centre de Recherche en Neurosciences de Lyon, Université Claude Bernard Lyon 1, Bron, France; ^2^International Institute for Integrative Sleep Medicine (WPI-IIIS), University of Tsukuba, Tsukuba, Japan; ^3^Graduate School of Medicine, Kyoto University, Kyoto, Japan; ^4^Faculty of Medicine, University of Tsukuba, Tsukuba, Japan

**Keywords:** sleep, REM sleep, EEG, imaging, species, evolution

## Abstract

Sleep is mandatory in most animals that have the nervous system and is universally observed in model organisms ranging from the nematodes, zebrafish, to mammals. However, it is unclear whether different sleep states fulfill common functions and are driven by shared mechanisms in these different animal species. Mammals and birds exhibit two obviously distinct states of sleep, i.e., non-rapid eye movement (NREM) sleep and rapid eye movement (REM) sleep, but it is unknown why sleep should be so segregated. Studying sleep in other animal models might give us clues that help solve this puzzle. Recent studies suggest that REM sleep, or ancestral forms of REM sleep might be found in non-mammalian or -avian species such as reptiles. These observations suggest that REM sleep and NREM sleep evolved earlier than previously thought. In this review, we discuss the evolutionary origin of the distinct REM/NREM sleep states to gain insight into the mechanistic and functional reason for these two different types of sleep.

## Introduction

Although a wealth of evidence suggests that sleep is related to many diverse processes such as memory consolidation, emotional stability and maintenance of brain homeostasis, the mechanisms by which sleep fulfills these functions are unclear (Rechtschaffen, 1998; Maquet, 2001; Siegel, 2003, 2005; Frank, 2006; Cirelli and Tononi, 2008; Walker, 2009, 2010; Schmidt, 2014). Even though unconsciousness in sleep is inexplicable, why did animals evolve the process of sleep rather than carrying out these functions under the protection of alert wakefulness? The emergence of sleep and wakefulness in various animal species likely depends on their living environments. The neuronal mechanisms and functions of these behaviors have thus likely evolved to adapt brain functions to each environment to support their behavior, maintain their nervous system and increase their fitness. In this context, wakefulness is responsible for behaviors that are directly related to motivation and fitness such as reproduction, foraging, and parental care. Likewise, sleep is a quiescent state, which is believed to be necessary for the maintenance of the nervous system and the body, including the system that governs consciousness, development, immunity, learning, and memory. Sleep architectures vary greatly among species, and extreme adaptation of sleep/wakefulness behavior to specific environments is observed (Campbell and Tobler, 1984; Lesku et al., 2006; Aulsebrook et al., 2016). For example, some marine mammals and birds have unique sleep behavior, i.e., unihemispheric sleep, during which one hemisphere generates NREM sleep-like activity, whereas the other side retains wakefulness (Mukhametov et al., 1977; Rattenborg et al., 2019), which allows animals to swim or fly while they are sleeping.

Besides behavioral criteria, the standard method to define sleep in animals with a well-developed cerebral cortex, like mammals and birds, is the observation of a pattern of coordinated changes in electroencephalogram (EEG) and electromyogram (EMG) readings. However, in other animals, sleep is usually defined solely by behavioral criteria (Campbell and Tobler, 1984): (1) stereotypic posture; (2) consolidated quiescence at a particular time of day; (3) increased arousal threshold; and (4) a compensatory increase in this behavior after deprivation.

**FIGURE 1:**
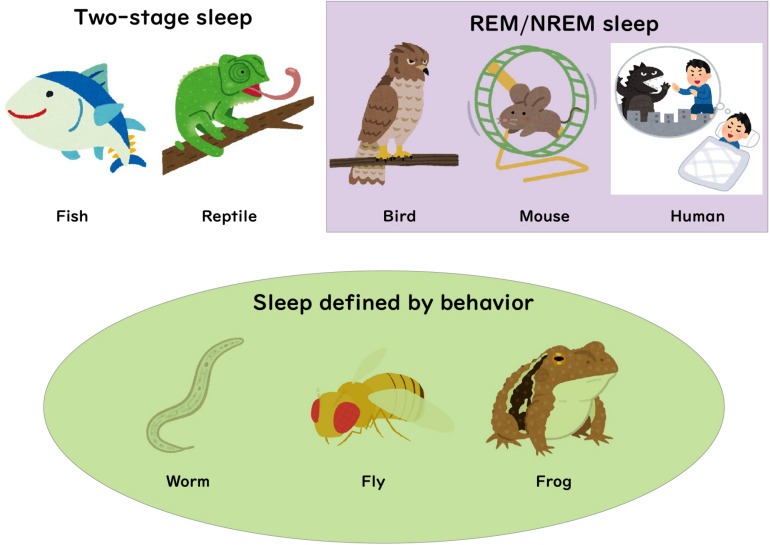
Sleep in various species. REM/NREM sleep states in mammals and birds are based on analysis of EEG/EMG recordings. Recent neuronal recording studies in fish and reptiles suggested the existence of two distinct states of sleep. Sleep states in other species including frogs, flies and worms have been measured using behavioral criteria.

In humans and various other mammalian species, NREM sleep and REM sleep stages are defined behaviorally and can be separated by EEG/EMG scoring (Figure 1). REM sleep as a distinct state was first discovered while observing children’s eye movements during sleep (Aserinsky and Kleitman, 1953). They discovered that 10–20 min episodes of rapid, jerky eye movements were interspersed among longer phases without ocular movement. Cortical activity during these phases resembled wake activity, challenging the notion of sleep as a uniformly low activity state (Loomis et al., 1937; Aserinsky, 1996). While the two states were observed in mammals and thereafter in birds, recent observations in reptiles indicate more convincingly than earlier studies that they could possess two electrophysiological sleep states (Shein-Idelson et al., 2016; Libourel et al., 2018). But what about other animal species? At which evolutional stage were REM and NREM sleep separated? In this review, we discuss how these two sleep states could have emerged during evolution.

## NREM and REM Sleep in Mammals and Birds

Observations about the existence of REM sleep in cats, rats and other mammals have provided fundamental information for understanding the architecture of sleep. Recent technological advances in the field of neuroscience, such as optogenetics and pharmacogenetics, as well as neuronal tracing techniques have accelerated our knowledge-gathering capabilities about the neuronal mechanisms that control sleep/wakefulness states in mammals (Scammell et al., 2017). Among mammalian species, mice have served as one of the most powerful model animals to examine the mechanisms and functions of sleep, in particular the neural circuits that regulate vigilance states. Inbred laboratory mouse strains have the same genetic backgrounds, their sleep/wakefulness states are easily observed by EEG/EMG recordings, and a large number of genetically modified strains are available. Nowadays, most of our detailed physiological data on sleep in mammals therefore stems from experiments on mice. Here we summarize knowledge that has been gained from them.

### Principles of NREM and REM Sleep

At least in mammals and birds, during wakefulness, the EEG shows a pattern of low amplitude, high frequency fluctuations, and the EMG shows considerable amounts of muscle activity. During NREM sleep, the EEG shows large amounts of oscillation in the delta (0–4 Hz) frequency ranges. Prolonged periods of wakefulness are usually followed by increased amounts of NREM sleep with high delta activity, showing the existence of compensatory and homeostatic mechanisms in its regulation (Borbély et al., 1984; Campbell, 2009).

After NREM sleep, the EEG starts to show abundant theta activity that arises from the dorsal hippocampus (Patel et al., 2012), which is start of the REM sleep. Anti-gravity muscle tone and body movements are mostly absent, because somatic motoneurons are inhibited by descending inhibitory pathways originating from the brainstem (Clément et al., 2011). In mammals, this is accompanied by characteristic rapid eye movements (REM) and fluctuations in heart rate and respiration; in species where it can be observed, whisking also occurs. In humans, subjects frequently experience emotional and story-like dreams during REM sleep (Hobson, 2009).

Since the neuronal regulation and the associated characteristics are very different, REM sleep might be highly distinct from NREM sleep. In rodents, under certain circumstances, the pressure to enter NREM sleep or REM sleep can build up and dissipate separately (Hayashi et al., 2015).

### Function of REM Sleep

The respective functions of NREM sleep and REM sleep are poorly understood and it is therefore not clear why these distinct states emerged during evolution. NREM sleep has been shown to be involved in several “housekeeping” processes of the brain, such as homeostatic synaptic plasticity, memory consolidation, and clearance of brain metabolites (Marshall et al., 2006; Rasch et al., 2007; Chauvette et al., 2012; Xie et al., 2013; Yang et al., 2014). Recent studies support the notion that REM sleep is also involved in memory consolidation (Boyce et al., 2016). Some functions of REM sleep seem to be related to the physiology and function of NREM sleep. Our recent work suggests that REM sleep serves to promote slow wave activity in subsequent NREM sleep (Hayashi et al., 2015), which in turn likely consolidates NREM sleep.

Another prominent role of REM sleep might be to support brain maturation in the early stages of life. It is known that in humans and other mammals, REM sleep is most abundant in early development stage, in particular in the neonatal period (Roffwarg et al., 1966; Jouvet-Mounier et al., 1970). Moreover, infant mammals including humans exibit more myoclonic twitches of skeletal muscles, which occur exclusively during REM sleep and are highly distinct from movements during waking, than do adults (Jouvet-Mounier et al., 1970). Nowadays it is considered that the twitches send sensory feedback to contribute to development of the sensorimotor system (Blumberg et al., 2013, Blumberg et al., 2020), or even possibly play a role in cortico [the cortical whisker barrel field (S1-BF)]-hippocampal (CA1) coherence (Del Rio-Bermudez et al., 2020), which could support later-emerging needs including spatial navigation. Spontaneous neuronal activity has crucial roles in various neuronal events that accompany brain maturation, ranging from neuronal differentiation, migration and myelination to synapse formation and elimination (Kirischuk et al., 2017). The wide activation of neurons during REM sleep might provide spontaneous patterns of neuronal activity suited for brain maturation.

The time spent in REM sleep varies not only during an individual’s development but also among species, as has been reported at least in mammals and birds (Allada and Siegel, 2008; Lesku et al., 2009; Siegel, 2009). For example, horses, giraffes and elephants spend less than 1 h in REM sleep each day, whereas ferrets, platypuses and house cats can spend 3 to 8 h per day in REM sleep. This is a key to better understanding the function of REM sleep, and thus there have been several attempts to identify factors that correlate with sleep time (e.g., body mass, risk of predation) (Lesku et al., 2009).

The existence of two distinct sleep states only in endotherms has been thought to be the other clue to understanding the evolution of sleep (Kavanau, 2002). For example, the decline in brain temperature during NREM sleep is reversed during REM sleep (Deboer et al., 1994), and thermoregulatory mechanisms during REM sleep are more limited (absence of piloerection and shivering, while more warm blood is pumped toward the brain from the core body as well as more blood flow), suggesting a possible relationship between thermoregulatory mechanisms and REM sleep function. Interestingly, during unihemispheric sleep, birds (great frigatebirds) and fur seals exhibit very low amounts of REM sleep (Rattenborg et al., 2016; Lyamin et al., 2018). One simple interpretation is that at least in these environments (water)/conditions (flying), full REM sleep might be too dangerous, and something like unihemispheric REM sleep could possibly work. But also, this suggests that functions of REM sleep might be related to changes in the brain that occur during global NREM sleep, such as a decrease in brain temperature and cortical function. Consistently, REM sleep is usually not observed directly after wakefulness, when brain temperature is considerably elevated. In this scheme, REM sleep periodically generates active states of the brain without mobilizing the system that governs alertness to maintain brain temperature and associated brain processes. However, more recent studies suggesting that a REM sleep-like state could exist in ectotherms, such as reptiles, teleost fishes, or even cuttlefish, cast major doubt on the hypothesis that REM sleep co-evolved with homeothermia (Frank et al., 2012; Shein-Idelson et al., 2016; Libourel et al., 2018; Iglesias et al., 2019; Leung et al., 2019).

## Sleep in Reptiles

Non–avian reptiles (lizards, snakes, turtles, tortoises, and crocodiles) are at key evolutionary positions, they are ectotherms, and share a common ancestor with mammals and birds, and thus can serve as precious model animals to decipher the evolution of neural functions – including sleep.

### Behavioral Sleep in Reptiles

Identifying sleep in reptiles is challenging mainly for two reasons. Firstly, they generally exhibit long periods of quiescence. Secondly, their cortical EEG does not allow unambiguous identification of sleep states (Libourel and Herrel, 2016). Quiescence is likely abundant because they are ectotherms and tend to minimize energy expenditure, spending most of their time waiting for prey, basking, or resting. However, behavioral sleep in reptiles is distinct from simple rest as they display a stereotypic position in a specific location, for example, curling into a shelter, lying with their head on a leaf, etc. Eye closure is not an indicator of sleep because the eyes are closed during basking, and some reptiles keep one eye open during behavioral sleep, although whether unihemispheric sleep exists in reptiles remains unknown (Kelly et al., 2015). Decreased respiratory and cardiac activities are characteristic of sleep in non-avian reptiles (Libourel and Herrel, 2016). Additionally, a high arousal threshold and homeostatic regulation have also been measured in various reptiles. The cortical EEG in reptiles does not provide any clear signatures of vigilance states (Libourel and Herrel, 2016). However, recent findings have provided new information suggesting that different sleep states could exit in lizards (Libourel and Herrel, 2016; Shein-Idelson et al., 2016).

#### Sleep State 1 – A NREM Sleep-Like State in Reptiles

While a consensus has not been reached as to whether reptilian homologs of NREM sleep and REM sleep exist, many studies have shown that high amplitude sharp waves appear during behavioral sleep, a fact that was pointed out in the 1990s (Libourel and Herrel, 2016). The Australian bearded dragon (*Pogona vitticeps*) exhibits a period of behavioral sleep rich in delta frequency (0.5–4 Hz) characterized by numerous negative high amplitude sharp waves (Shein-Idelson et al., 2016). This activity was detected from the dorsal ventricular ridge (DVR), a possible homolog of the mammalian isocortex, the amygdala, and/or claustral complex (Aboitiz et al., 2002; Tosches et al., 2018). This sleep state was proposed by the authors to be homologous to NREM sleep, because NREM sleep is characterized by cortical slow waves (delta waves) and hippocampal sharp-wave ripples. The Argentine tegu (*Salvator merianae*) also exhibits high voltage sharp waves occurring during behavioral sleep in some brain regions including the DVR (Libourel et al., 2018). However, cautious interpretation is necessary as to whether the sleep states in the two species are homologous to each other, as the sharp waves have different properties of density and duration: the morphological characters observed in the bearded dragon (occurring at a high rate of 60–120 per min with a half width of 100–400 ms) were rather similar to mammalian slow waves, while ones in the Argentine tegu (lasting less than 50 ms with an amplitude of 0.2–1 mV, appearing mostly during sleep state 1 at a rate of 1 per min) resembled to the mammalian hippocampal sharp waves. Sharp waves are also detected in turtles and crocodiles (Kelly et al., 2015; Tisdale et al., 2018). It is still not clear if these “sharp waves” in reptiles are homologous to mammalian hippocampal sharp-wave ripple complexes or cortical slow waves that occur during NREM sleep. Supporting the argument of homology to NREM sleep, reptilian sharp waves are suggested to be a marker of homeostatic sleep pressure just like slow waves in mammals (Flanigan, 1973, 1974; Flanigan et al., 1974; Libourel and Herrel, 2016). In addition, reptilian sharp waves can propagate across the brain (Norimoto et al., 2020), similarly to slow waves in mammals and birds (Massimini et al., 2004; Beckers and Rattenborg, 2015). Recent studies have shown that the anterior medial pole of the DVR (amDVR) and the pallial thickening in the bearded dragon and turtle *Trachemys script*, respectively, are possible homologs of the mammalian claustrum, and that the amDVR plays a crucial role in generating the sharp waves during sleep in the bearded dragon (Tosches et al., 2018; Norimoto et al., 2020). Notably, in mammals, some neuronal populations in the claustrum promote the generation of slow waves (Narikiyo et al., 2020). Altogether, there are some similarities between reptilian sleep with sharp waves and NREM sleep. However, whether these two states support the same function remains unknown, and further investigations are needed to reach a clear conclusion on their common evolutionary origins.

#### Sleep State 2 – A REM Sleep-Like State in Reptiles

In mammals and birds, REM sleep is characterized by features such as wake-like brain activity, muscle atonia, REMs, and muscle twitches. In addition, homeostatic changes including a decrease in thermoregulation occur. Several previous studies, for example in the green iguana (Ayala-Guerrero and Mexicano, 2008), suggested the existence of REM sleep-like states in reptiles, based on the occurrence of eye movements associated with wake-like EEG activity and/or involuntary movements. However, the possibility that the animals are in a briefly awakened state rather than a REM sleep-like sleep state could not be ruled out (Libourel and Herrel, 2016). In the bearded dragon and the Argentine tegu, sleep state 1, which is rich in sharp waves, alternates with another sleep-like state, just like mammalian sleep alternates between NREM sleep and REM sleep. During this sleep state 2, in the DVR, it was shown that β waves (10–40 Hz) and dominant oscillations at 15 Hz frequency were present, in the Australian dragon and the Argentine tegu, respectively (Shein-Idelson et al., 2016; Libourel et al., 2018). The 15 Hz oscillation in the tegu was not detected during wakefulness, suggesting that the state is distinct from wakefulness. Both species tended to show eye movements associated with these brain activities. Moreover, fluoxetine treatment, which suppresses REM sleep in mammals, suppressed the 15 Hz oscillation in the tegu, further supporting the commonality between the two states (Libourel et al., 2018). However, clear signs of muscle atonia or twitches were not detected.

### Sleep Architecture and Mechanisms of Sleep-Wake Cycles in Reptiles

In the Argentine tegu, the time spent in sleep state 1 is about 6 times longer than that in sleep state 2, and sleep state 2 tends to occur preferentially at the beginning or at the end of the dark period (Libourel et al., 2018). In contrast, the time spent in each sleep state in the Australian bearded dragon is almost the same (Shein-Idelson et al., 2016). In the Australian bearded dragon, the two states alternate regularly throughout the night with a period of about 80–90 s. The cycle of these alternations is affected by the environmental temperature; it is prolonged or shortened when the temperature declines or rises, respectively. Why the cycle and duration of each state differ among reptilian species remains unclear, but this is also true for mammalian NREM-REM sleep as mentioned in section “NREM and REM Sleep in Mammals and Birds.” Likewise, cycles of REM sleep (inter-REM intervals) also substantially differ across animal species. In mice, REM sleep occurs every 10–15 min, while in humans, REM sleep occurs every 70–120 min.

The mechanisms underlying switching from one sleep state to the other, or from arousal to sleep in reptiles are unknown. However, some of the neuropeptides, neuromodulators and brain structures that are important for regulating arousal and REM sleep in mammals are also found in reptiles. For example, the neuropeptide orexin, which in mammals is produced in the lateral hypothalamus and is crucial for maintaining arousal (Sakurai, 2007), is produced in the periventricular hypothalamic nucleus and infundibular hypothalamus in the gecko and pseudemis (Domínguez et al., 2010). These orexinergic neurons in reptiles innervate the substantia nigra and ventral tegmental area in the midbrain tegmentum, and the locus coeruleus, nucleus of the solitary tract, and raphe nuclei in the brainstem (Domínguez et al., 2010).

Mammalian REM sleep is generated and maintained by multiple nuclei in the brainstem and hypothalamus, which are highly conserved across vertebrates. In mammals, melanin-concentrating hormone producing neurons (MCH neurons) located in the lateral hypothalamus are important in maintaining and generating REM sleep. In reptiles, MCH neurons reside in the periventricular and lateral hypothalamic area (Cardot et al., 1994). In mice, Lhx6-expressing neurons in the zona incerta are activated during REM sleep and are involved in regulating sleep (Liu et al., 2017; Lee et al., 2020). Lhx6 is a LIM homeodomain factor which is conserved in chicken and anuran (Medina et al., 2011), and thus likely also in reptiles. Further investigations are needed to address whether these conserved factors have equivalent roles in sleep-wakefulness cycle control in reptiles as in mammals.

### Comparing Physiology and Anatomy of Reptile and Mammalian Sleep

Brain temperature declines during NREM sleep and rises during REM sleep in endotherms (Deboer et al., 1994). We still do not have a clear answer on whether this feature is conserved in the two sleep states of reptiles as they are in ectotherms. In addition, while mammals and birds show clear changes in muscle tone depending on the vigilance state, in reptiles it seems to always be reduced or atonic during resting. Furthermore, careful interpretation of the EEG or local field potentials (LFPs) is necessary, as the frequency and amplitude of EEG fluctuations are affected by temperature. From an anatomical point of view, the cortex in reptiles consists of only three layers and lacks the counterparts of mammalian layers II and III, which are important in generating slow waves. Considering these differences, even though reptiles have two sleep states, each sharing some features with NREM and REM sleep, their physiological roles might be different.

## Sleep in Non-Amniotic Vertebrates

In zebrafish, sleep has been defined solely by behavioral criteria based on periods of quiescence associated with a specific posture (either floating with head down or staying in a horizontal position close to the bottom of the chamber) (Zhdanova et al., 2001), as it is difficult to score vigilance states by electrophysiological criteria in the fish brain due to the absence of a conventional neocortex.

### Two Distinct Neuronal States Observed in Zebrafish During Sleep

A recent study suggested that there are at least two sleep states in fish (Leung et al., 2019). The research group performed fluorescent calcium imaging in the dorsal pallidum in larval zebrafish, revealing dynamic activity pattern changes during sleep and wakefulness (Leung et al., 2019). In awake fish, high spontaneous and de-synchronous activity in the dorsal pallium was observed, while highly synchronous bursts of activity were observed in the same region during sleep. Synchronous oscillatory neuronal states during sleep resembled cortical activity during NREM sleep in mammals. Furthermore, sleep deprivation or a histamine H1R antagonist increased this NREM sleep-like state, underscoring the similarity between zebrafish activity and mammalian NREM sleep.

Moreover, the authors reported that they observed two distinct sleep states in zebrafish by analyzing eye movement, heart and muscle activity, along with brain-wide activity imaging. The authors termed these two states “slow bursting sleep” and “propagating wave sleep,” which share similarities with mammalian NREM and REM sleep. Notably, propagating wave sleep was accompanied by increased variance in the distribution of inter-heartbeat intervals, showing similarity to mammalian REM sleep. These observations suggest that two states of sleep are found even in non-amniotic vertebrates. Furthermore, MCH was shown to regulate propagating wave sleep in zebrafish, similarly to the effect of MCH on REM sleep in mice, which also suggests similarity between propagating wave sleep and mammalian REM sleep. This study revealed that a REM sleep-like state exists even in distantly related vertebrates. Since REM sleep is thought to be important in the development of the brain, it is important to know whether adult fish also show a REM sleep-like state.

## Sleep in Invertebrates

### Cephalopods – Common Cuttlefish

What about sleep in invertebrates? The common cuttlefish (*Sepia Officinalis*), one of the cephalopods that has the largest and most complex brain (Nixon and Young, 2003), has been shown to possess two different sleep-like states: a quiescent sleep state and a REM-sleep like state (Frank et al., 2012; Iglesias et al., 2019). Frank and Iglesias examined sleep in juveniles, senescent adults, and non-senescent adults. The common cuttlefish spends about one-third of the day sleeping, 7% of which is a REM-sleep like state, characterized by general immobility with occasional twitching, REM, and rapid skin pattern changes including specific darkening around the eyes (Frank et al., 2012; Iglesias et al., 2019). Interestingly, a sleep state with these features was not observed in juveniles (Frank et al., 2012), which opposes what we have seen in mammals. Considering that they seem to be at high risk during this state, it is surprising that they spend more time in this REM sleep-like state than do some marine mammals and birds. Still, the reason and meaning of having this state in cuttlefish, and whether other cephalopods also possess this state are unknown. Therefore, further investigation and careful interpretation concerning the recording environment are needed.

### Fruit Fly and Nematode – Powerful Models for Understanding Genetic Regulation of Sleep

Among several model animals used to study sleep in invertebrates, the fruit fly (*Drosophila)* is particularly useful to study at a molecular level. It is suitable for non-biased screens to decipher the genetic components underlying unidentified biological phenomena because of its short reproductive cycle and the availability of genetic tools. Consequently, many findings about sleep have been obtained through studies on flies. The fly sleep model is now widely accepted and has inspired the development of other genetic models of sleep, such as the nematode (*C. elegans*) (Raizen et al., 2008). The use of these models has rapidly provided insights into the mechanisms of sleep in other animals.

It is not clear whether flies and nematodes also display dynamic neuronal processes such as REM and NREM sleep because their sleep has been solely defined by behavioral criteria. Recently, it was reported that a gene coding salt-inducible kinase 3 (SIK3) positively regulates NREM sleep in mice (Funato et al., 2016). Interestingly, this gene is not only widely conserved from invertebrates including flies and nematodes to mammals, but also seems to play similar roles in invertebrates, because loss of function decreased total sleep time in flies and nematodes (Funato et al., 2016). This finding has impacted on the sleep research field since it indicated the possibility that NREM sleep, but not REM sleep, is an evolutionarily conserved sleep state, although further careful research is required. In addition, fly sleep seems to serve similar functions to those in mammals such as synaptic downscaling and memory consolidation. Also, since the fly brain is comprised of more than 100,000 neurons, synchronicity of their firing might be a clue as to whether fly sleep also contains distinct states. Further development of electrophysiological measurements and/or functional imaging techniques of the whole fly brain may enable us to define sleep in flies by neuronal activities, revealing whether fly sleep has distinct stages, and serve as a good platform to understand the function of REM sleep.

## Conclusion

It is likely that some reptiles, teleosts, and even cuttlefish have two different electrophysiological sleep states, sharing some features with NREM and REM sleep in mammals. However, it should be noted that mammalian NREM and REM sleep have been defined based on measurable traits constrained by anatomical or physiological features specific to mammals (Blumberg et al., 2020). Thus, applying these criteria to sleep states in other species that have very different neuroanatomy, metabolism, and lifestyle still remains controversial. Thus, the evolutionary origin of NREM and REM sleep would find answers only if we can draw a bigger picture of sleep, perhaps by thinking outside mammalian-centered definitions and identifying more traits associated with the sleep phenotype in a more diverse array of animal species (Blumberg et al., 2020). Understanding the factors involved in the regulation of sleep in various species might be a helpful start. Acetylcholine and monoamines, including noradrenaline, serotonin, histamine and dopamine, play important roles in regulating sleep/wakefulness states and behavior in mammals (Scammell et al., 2017). These factors are also important in lower vertebrates and invertebrates. Neuropeptides such as orexins and MCH also play a role in regulation of sleep/wakefulness states in mammals and other species (Sakurai, 2014). Especially, orexins are essential for the maintenance of long, consolidated wakefulness, which is necessary for performing any purposeful behaviors. Orexin-induced arousal is regulated via noradrenaline signaling in zebrafish (Singh et al., 2015). All these factors are relatively strongly conserved in evolution. Phylogenetic aspects of these factors might help us understand the evolution of sleep of vertebrates.

Recent studies showing there are two distinct states of sleep in reptiles fish and cuttlefish have opened up avenues of research toward understanding REM sleep, although we are only half-way down this road. It remains to be elucidated why the brain needs two states of sleep, i.e., sleep with highly synchronous and de-synchronized firing of neurons.

## Summary

Sleep is essential in most animals that have the nervous system, and is universally observed in model animals ranging from nematodes to zebrafish to mammals. Mammals and birds exhibit two obviously distinct states of sleep, i.e., non-rapid eye movement (NREM) sleep and rapid eye movement (REM) sleep, but it is unknown why sleep should be so segregated. In addition, it is unclear whether the different sleep states fulfill common functions and are driven by shared mechanisms in these different animal species. Studying sleep in other animal models might give us clues that could help solve this puzzle. Recent studies suggest that REM sleep or ancestral forms of REM sleep might be found in non-mammalian and non-avian species such as reptiles. These observations suggest that REM sleep and NREM sleep evolved earlier than previously thought. In this review, we discuss the evolutionary origin of the distinct REM/NREM sleep states to gain an insight into the mechanistic and functional reasons for these two different types of sleep.

## Author Contributions

All authors listed have made a substantial, direct and intellectual contribution to the work, and approved it for publication.

## Conflict of Interest

The authors declare that the research was conducted in the absence of any commercial or financial relationships that could be construed as a potential conflict of interest.
